# Supplementary material for: Learning to detect AI texts and learning the limits
Source: PLoS One. 2025 Oct 15;20(10):e0333007. doi: 10.1371/journal.pone.0333007 (PMC12527182; doi:10.1371/journal.pone.0333007)
Supplement: S2 File — All data and scripts used in the study, some additional descriptive statistics, scalable formate figures. (ZIP) [file pone.0333007.s002.tgz › data and scripts/OSF/scripts/analysis/mixed models jamovi/outputs/complete_correctness_learners.pdf]

Generalized Mixed Model

Model Info

Info	Value	Comment
Model Type	Logistic	Model for binary y
Call	glm	Is correct ~ 1 + More readable is human + Confidence + Demographics: age + Demographics: education_ordinal + Demographics: ai_usage_ordinal + Demographics: gender merged + Demographics: field_merged + Reaction time log + GLS Euclidean distance + GLS1 difference + GLS2 difference + GLS3 difference + GLS4 difference + id + Demographics: reading_disorder_merged + GLS8 difference + GLS7 difference + GLS6 difference + GLS5 difference + Demographics: ai_improve + Demographics: ai_risk + Demographics: ai_quality + Demographics: ai_lacks_creativity + Demographics: ai_lacks_understanding + Demographics: ai_grammar + Demographics: ai_avoidance + Demographics: ai_dumbing + Demographics: ai_fascination + (0 + More readable is human + Confidence + Reaction time log + GLS Euclidean distance + GLS1 difference + GLS2 difference + GLS3 difference + GLS4 difference + id + GLS8 difference + GLS7 difference + GLS6 difference + GLS5 difference | Participant ID)
Link function	Logit	Log of the odd of y=1 over y=0
Direction	P(y=1)/P(y=0)	P(Is correct = 1) / P(Is correct = 0)
Distribution	Binomial	Dichotomous event distribution of y
LogLikel.	-1173.143	Unconditional Log-Likelihood
-2*LogLikel.	2346.287	Unconditional absolute deviance
Deviance	1705.411	Conditional relative deviance
R-squared	NaN	Marginal
R-squared	NaN	Conditional
AIC	2620.290	Less is better
BIC	3392.588	Less is better
Residual DF	1937.000	
Chi-squared/DF	0.675	Overdispersion indicator
Converged	yes	
Optimizer	bobyqa	

Note. R-squared cannot be computed.

Note. boundary (singular) fit: see ?isSingular

Model Results

Fixed Effect Omnibus tests

	X^2	df	p
More readable is human	19.46880	1.00	< .001
Confidence	2.95358	1.00	0.086
Demographics: age	0.13244	1.00	0.716
Demographics: education_ordinal	0.29586	1.00	0.586
Demographics: ai_usage_ordinal	0.69590	1.00	0.404
Demographics: gender merged	0.15981	1.00	0.689
Demographics: field_merged	10.29144	4.00	0.036
Reaction time log	0.02321	1.00	0.879
GLS Euclidean distance	0.00343	1.00	0.953
GLS1 difference	0.59204	1.00	0.442
GLS2 difference	3.41109	1.00	0.065
GLS3 difference	2.16118	1.00	0.142
GLS4 difference	0.67797	1.00	0.410
id	16.08902	1.00	< .001
Demographics: reading_disorder_merged	1.71524	1.00	0.190
GLS8 difference	0.17682	1.00	0.674
GLS7 difference	0.79916	1.00	0.371
GLS6 difference	4.06814	1.00	0.044
GLS5 difference	0.22886	1.00	0.632
Demographics: ai_improve	1.57010	1.00	0.210
Demographics: ai_risk	0.03050	1.00	0.861
Demographics: ai_quality	2.11651	1.00	0.146
Demographics: ai_lacks_creativity	0.45344	1.00	0.501
Demographics: ai_lacks_understanding	1.15976	1.00	0.282
Demographics: ai_grammar	0.06892	1.00	0.793
Demographics: ai_avoidance	5.27193	1.00	0.022
Demographics: ai_dumbing	0.04171	1.00	0.838
Demographics: ai_fascination	0.03826	1.00	0.845

Fixed Effects Parameter Estimates

Names	Effect	Estimate	SE	95% Confidence Interval		exp(B)	95% Exp(B) Confidence Interval		z	p
				Lower	Upper		Lower	Upper		
(Intercept)	(Intercept)	1.75347	0.3499	1.06769	2.4393	5.775	2.9087	11.464	5.0114	< .001
More readable is human1	1 - 0	1.22118	0.2768	0.67873	1.7636	3.391	1.9714	5.834	4.4123	< .001
Confidence	Confidence	0.08892	0.0517	-0.01249	0.1903	1.093	0.9876	1.210	1.7186	0.086
Demographics: age	Demographics: age	0.00393	0.0108	-0.01723	0.0251	1.004	0.9829	1.025	0.3639	0.716
Demographics: education_ordinal	Demographics: education_ordinal	-0.07256	0.1334	-0.33404	0.1889	0.930	0.7160	1.208	-0.5439	0.586
Demographics: ai_usage_ordinal	Demographics: ai_usage_ordinal	0.05217	0.0625	-0.07040	0.1747	1.054	0.9320	1.191	0.8342	0.404
Demographics: gender merged1	male - female	-0.08266	0.2068	-0.48794	0.3226	0.921	0.6139	1.381	-0.3998	0.689
Demographics: field_merged1	human - cs	-1.70755	0.8003	-3.27602	-0.1391	0.181	0.0378	0.870	-2.1338	0.033
Demographics: field_merged2	nature - cs	-1.68028	0.8012	-3.25057	-0.1100	0.186	0.0388	0.896	-2.0972	0.036
Demographics: field_merged3	other - cs	-1.27117	0.8850	-3.00583	0.4635	0.281	0.0495	1.590	-1.4363	0.151
Demographics: field_merged4	philology - cs	-1.17388	0.7908	-2.72376	0.3760	0.309	0.0656	1.456	-1.4845	0.138
Reaction time log	Reaction time log	0.01424	0.0935	-0.16893	0.1974	1.014	0.8446	1.218	0.1523	0.879
GLS Euclidean distance	GLS Euclidean distance	-0.00341	0.0581	-0.11732	0.1105	0.997	0.8893	1.117	-0.0586	0.953
GLS1 difference	GLS1 difference	-0.08284	0.1077	-0.29387	0.1282	0.920	0.7454	1.137	-0.7694	0.442
GLS2 difference	GLS2 difference	-0.28056	0.1519	-0.57830	0.0172	0.755	0.5608	1.017	-1.8469	0.065
GLS3 difference	GLS3 difference	0.06608	0.0449	-0.02202	0.1542	1.068	0.9782	1.167	1.4701	0.142
GLS4 difference	GLS4 difference	0.04319	0.0525	-0.05962	0.1460	1.044	0.9421	1.157	0.8234	0.410
id	id	0.05304	0.0132	0.02712	0.0790	1.054	1.0275	1.082	4.0111	< .001
Demographics: reading_disorder_merged1	true - false	0.72202	0.5513	-0.35851	1.8025	2.059	0.6987	6.065	1.3097	0.190
GLS8 difference	GLS8 difference	0.01752	0.0417	-0.06414	0.0992	1.018	0.9379	1.104	0.4205	0.674
GLS7 difference	GLS7 difference	-0.04552	0.0509	-0.14532	0.0543	0.956	0.8647	1.056	-0.8940	0.371
GLS6 difference	GLS6 difference	0.07922	0.0393	0.00224	0.1562	1.082	1.0022	1.169	2.0170	0.044
GLS5 difference	GLS5 difference	-0.03025	0.0632	-0.15419	0.0937	0.970	0.8571	1.098	-0.4784	0.632
Demographics: ai_improve	Demographics: ai_improve	0.09082	0.0725	-0.05124	0.2329	1.095	0.9501	1.262	1.2530	0.210
Demographics: ai_risk	Demographics: ai_risk	0.01045	0.0598	-0.10683	0.1277	1.011	0.8987	1.136	0.1746	0.861
Demographics: ai_quality	Demographics: ai_quality	0.09781	0.0672	-0.03396	0.2296	1.103	0.9666	1.258	1.4548	0.146
Demographics: ai_lacks_creativity	Demographics: ai_lacks_creativity	0.04339	0.0644	-0.08290	0.1697	1.044	0.9204	1.185	0.6734	0.501
Demographics: ai_lacks_understanding	Demographics: ai_lacks_understanding	0.06126	0.0569	-0.05023	0.1728	1.063	0.9510	1.189	1.0769	0.282
Demographics: ai_grammar	Demographics: ai_grammar	-0.01654	0.0630	-0.14001	0.1069	0.984	0.8693	1.113	-0.2625	0.793
Demographics: ai_avoidance	Demographics: ai_avoidance	0.12247	0.0533	0.01793	0.2270	1.130	1.0181	1.255	2.2961	0.022
Demographics: ai_dumbing	Demographics: ai_dumbing	0.01103	0.0540	-0.09478	0.1168	1.011	0.9096	1.124	0.2042	0.838
Demographics: ai_fascination	Demographics: ai_fascination	0.01445	0.0739	-0.13032	0.1592	1.015	0.8778	1.173	0.1956	0.845

Random Components

Groups	Name	SD	Variance	ICC
Participant ID	More readable is human0	0.8707	0.75812	
	More readable is human1	1.6450	2.70616	
	Confidence	0.2783	0.07746	
	Reaction time log	0.2246	0.05044	
	GLS Euclidean distance	0.2031	0.04125	
	GLS1 difference	0.6851	0.46940	
	GLS2 difference	0.6633	0.43995	
	GLS3 difference	0.1631	0.02660	
	GLS4 difference	0.0705	0.00497	
	id	0.0590	0.00348	
	GLS8 difference	0.1607	0.02584	
	GLS7 difference	0.1277	0.01631	
	GLS6 difference	0.1719	0.02954	
	GLS5 difference	0.2500	0.06249	
Residuals		1.0000	1.00000	.

Note. Number of Obs: 2074 , groups: Participant ID 122

Random Parameters correlations

Groups	Param.1	Param.2	Corr.
Participant ID	More readable is human0	More readable is human1	-0.74893
	More readable is human0	Confidence	0.69299
	More readable is human0	Reaction time log	0.14039
	More readable is human0	GLS Euclidean distance	-0.11741
	More readable is human0	GLS1 difference	0.00524
	More readable is human0	GLS2 difference	-0.05658
	More readable is human0	GLS3 difference	0.16093
	More readable is human0	GLS4 difference	0.14308
	More readable is human0	id	0.39597
	More readable is human0	GLS8 difference	-0.05064
	More readable is human0	GLS7 difference	0.10114
	More readable is human0	GLS6 difference	0.32191
	More readable is human0	GLS5 difference	0.30439
	More readable is human1	Confidence	-0.36201
	More readable is human1	Reaction time log	-0.10605
	More readable is human1	GLS Euclidean distance	0.18802
	More readable is human1	GLS1 difference	0.07698
	More readable is human1	GLS2 difference	-0.43645
	More readable is human1	GLS3 difference	-0.04425
	More readable is human1	GLS4 difference	0.28439
	More readable is human1	id	-0.20016
	More readable is human1	GLS8 difference	0.09590
	More readable is human1	GLS7 difference	-0.08440
	More readable is human1	GLS6 difference	-0.14409
	More readable is human1	GLS5 difference	0.22300
	Confidence	Reaction time log	0.62084
	Confidence	GLS Euclidean distance	5.82e-4
	Confidence	GLS1 difference	0.13458
	Confidence	GLS2 difference	-0.32724
	Confidence	GLS3 difference	-0.16975
	Confidence	GLS4 difference	-0.02755
	Confidence	id	0.70806
	Confidence	GLS8 difference	-0.56421
	Confidence	GLS7 difference	0.27740
	Confidence	GLS6 difference	0.70559
	Confidence	GLS5 difference	0.43945
	Reaction time log	GLS Euclidean distance	-0.47991
	Reaction time log	GLS1 difference	0.33138
	Reaction time log	GLS2 difference	-0.31651
	Reaction time log	GLS3 difference	-0.23088
	Reaction time log	GLS4 difference	-0.15295
	Reaction time log	id	0.90143
	Reaction time log	GLS8 difference	-0.85447
	Reaction time log	GLS7 difference	0.29589
	Reaction time log	GLS6 difference	0.86667
	Reaction time log	GLS5 difference	0.18886
	GLS Euclidean distance	GLS1 difference	-0.42465
	GLS Euclidean distance	GLS2 difference	0.31367
	GLS Euclidean distance	GLS3 difference	-0.41959
	GLS Euclidean distance	GLS4 difference	-0.36307
	GLS Euclidean distance	id	-0.63713
	GLS Euclidean distance	GLS8 difference	0.30621
	GLS Euclidean distance	GLS7 difference	0.09862
	GLS Euclidean distance	GLS6 difference	-0.48188
	GLS Euclidean distance	GLS5 difference	0.02764
	GLS1 difference	GLS2 difference	-0.57781
	GLS1 difference	GLS3 difference	0.57626

Random Parameters correlations

Groups	Param.1	Param.2	Corr.
	GLS1 difference	GLS4 difference	0.46697
	GLS1 difference	id	0.29781
	GLS1 difference	GLS8 difference	-0.64415
	GLS1 difference	GLS7 difference	0.52507
	GLS1 difference	GLS6 difference	0.66018
	GLS1 difference	GLS5 difference	0.49995
	GLS2 difference	GLS3 difference	-0.57099
	GLS2 difference	GLS4 difference	-0.81916
	GLS2 difference	id	-0.47639
	GLS2 difference	GLS8 difference	0.35691
	GLS2 difference	GLS7 difference	0.09307
	GLS2 difference	GLS6 difference	-0.49190
	GLS2 difference	GLS5 difference	-0.48264
	GLS3 difference	GLS4 difference	0.85285
	GLS3 difference	id	-0.00918
	GLS3 difference	GLS8 difference	0.03398
	GLS3 difference	GLS7 difference	-0.26463
	GLS3 difference	GLS6 difference	0.03420
	GLS3 difference	GLS5 difference	0.04830
	GLS4 difference	id	0.13398
	GLS4 difference	GLS8 difference	0.10999
	GLS4 difference	GLS7 difference	-0.30214
	GLS4 difference	GLS6 difference	0.09777
	GLS4 difference	GLS5 difference	0.33781
	id	GLS8 difference	-0.67682
	id	GLS7 difference	0.09342
	id	GLS6 difference	0.84847
	id	GLS5 difference	0.25491
	GLS8 difference	GLS7 difference	-0.51185
	GLS8 difference	GLS6 difference	-0.88612
	GLS8 difference	GLS5 difference	-0.25024
	GLS7 difference	GLS6 difference	0.54724
	GLS7 difference	GLS5 difference	0.66339
	GLS6 difference	GLS5 difference	0.53784
